# Explanation and Elaboration with Examples for CLEAR (CLEAR-E3): an EuSoMII Radiomics Auditing Group Initiative

**DOI:** 10.1186/s41747-024-00471-z

**Published:** 2024-05-14

**Authors:** Burak Kocak, Alessandra Borgheresi, Andrea Ponsiglione, Anna E. Andreychenko, Armando Ugo Cavallo, Arnaldo Stanzione, Fabio M. Doniselli, Federica Vernuccio, Matthaios Triantafyllou, Roberto Cannella, Romina Trotta, Samuele Ghezzo, Tugba Akinci D’Antonoli, Renato Cuocolo

**Affiliations:** 1 Department of Radiology, University of Health Sciences, Basaksehir Cam and Sakura City Hospital, Basaksehir, Istanbul, Turkey; 2 Department of Clinical, Special and Dental Sciences, University Politecnica delle Marche, Ancona, Italy; 3 Department of Radiology, University Hospital “Azienda Ospedaliero Universitaria delle Marche”, Via Conca 71, 60126 Ancona, Italy; 4 Department of Advanced Biomedical Sciences, University of Naples Federico II, Naples, Italy; 5 Laboratory for Digital Public Health Technologies, ITMO University, St. Petersburg, Russian Federation; 6 Division of Radiology, Istituto Dermopatico dell’Immacolata (IDI) IRCCS, Rome, Italy; 7 Neuroradiology Unit, Fondazione Istituto Neurologico Carlo Besta, Via Celoria 11, 20133 Milano, Italy; 8 Section of Radiology, Department of Biomedicine, Neuroscience and Advanced Diagnosis (Bi.N.D), University of Palermo, 90127 Palermo, Italy; 9 Department of Medical Imaging, University Hospital of Heraklion, 71110 Crete, Voutes Greece; 10 Section of Radiology - Department of Biomedicine, Neuroscience and Advanced Diagnostics (BiND), University of Palermo, Palermo, Italy; 11 Department of Radiology - Fatima Hospital, Seville, Spain; 12 Vita-Salute San Raffaele University, Milan, Italy; 13 Institute of Radiology and Nuclear Medicine, Cantonal Hospital Baselland, Liestal, Switzerland; 14 Department of Medicine, Surgery and Dentistry, University of Salerno, Baronissi, Italy

**Keywords:** Checklist, Guideline, Machine learning, Radiomics, Reporting

## Abstract

Overall quality of radiomics research has been reported as low in literature, which constitutes a major challenge to improve. Consistent, transparent, and accurate reporting is critical, which can be accomplished with systematic use of reporting guidelines. The CheckList for EvaluAtion of Radiomics research (CLEAR) was previously developed to assist authors in reporting their radiomic research and to assist reviewers in their evaluation. To take full advantage of CLEAR, further explanation and elaboration of each item, as well as literature examples, may be useful. The main goal of this work, Explanation and Elaboration with Examples for CLEAR (CLEAR-E3), is to improve CLEAR’s usability and dissemination. In this international collaborative effort, members of the European Society of Medical Imaging Informatics−Radiomics Auditing Group searched radiomics literature to identify representative reporting examples for each CLEAR item. At least two examples, demonstrating optimal reporting, were presented for each item. All examples were selected from open-access articles, allowing users to easily consult the corresponding full-text articles. In addition to these, each CLEAR item’s explanation was further expanded and elaborated. For easier access, the resulting document is available at https://radiomic.github.io/CLEAR-E3/. As a complementary effort to CLEAR, we anticipate that this initiative will assist authors in reporting their radiomics research with greater ease and transparency, as well as editors and reviewers in reviewing manuscripts.

**Relevance statement **Along with the original CLEAR checklist, CLEAR-E3 is expected to provide a more in-depth understanding of the CLEAR items, as well as concrete examples for reporting and evaluating radiomic research.

**Key points**

• As a complementary effort to CLEAR, this international collaborative effort aims to assist authors in reporting their radiomics research, as well as editors and reviewers in reviewing radiomics manuscripts.

• Based on positive examples from the literature selected by the EuSoMII Radiomics Auditing Group, each CLEAR item explanation was further elaborated in CLEAR-E3.

• The resulting explanation and elaboration document with examples can be accessed at https://radiomic.github.io/CLEAR-E3/.

**Graphical Abstract:**

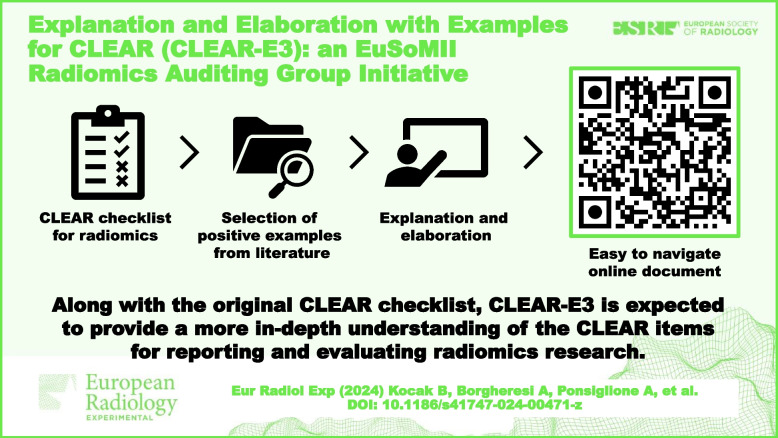

## Introduction

Radiomics is a collection of quantitative medical image analysis methods based on sophisticated mathematical approaches for extraction and analysis of biomarkers (*i.e.*, features) to enhance the information currently accessible to physicians [1]. The key premise of radiomics is that disease-specific complex patterns and biological insights are present in medical images but undetectable to the human eye. These are usually revealed with the use of machine learning techniques [2, 3]. Through various hand-crafted and deep learning-based radiomic approaches, numerous research studies have demonstrated encouraging results for a range of prediction tasks, such as diagnosis [4], genomics [5], and clinical outcomes [6, 7].

As of March 2024, a simple search for “radiomics” in PubMed returns around 10,800 papers, of which more than half were published after 2022. This finding provides further evidence that the radiomics literature has been expanding at an exponential rate, with a reported annual growth rate of 29.1% and a doubling time of 2.7 years for the time period between 2017 and 2021 [8]. Despite such an increase in research output over recent years, the vast majority of radiomic studies unfortunately failed to be clinically useful, which is also clearly noticeable in the US Food and Drug Administration clearance of tools related to radiomics [9, 10]. Similarly, a recent overview of the meta-analyses concluded that more evidence is still needed to support the clinical translation of radiomics research [11]. Interestingly, despite these shortcomings, papers with positive results continue to heavily predominate in the radiomics literature [12–14], with very few studies presenting negative results [15, 16].

The current lack of clinical translation might be attributable to the fact that radiomics is a complex multi-step process that leads to numerous challenges related to robustness, reproducibility, replicability, and generalizability [17–19]. The key to bridging the current translational gap between exploratory radiomics research and clinically validated decision-making tools may lie in the standardization of the radiomics pipeline including its reporting [9, 20]. With its recent extended effort on convolutional filters [21], the Image Biomarker Standardisation Initiative (IBSI) has been a significant driving force to standardize feature computation-related aspects [22]. Regarding transparent reporting and methodological quality evaluation, new consensus guidelines have been published and endorsed by prominent imaging societies [23, 24].

Lack of shared standards and practices in radiomics, especially in the years following its introduction in the research domain may have fostered poor methodological quality [25]. On the other hand, increased leeway in study design might be considered acceptable to some level to develop or test novel methods that can be more robustly validated by future research. Transparent reporting via a systematic approach (*i.e.*, based on established guidelines) has been considered essential to improve the reproducibility of scientific research [26]. According to a recent metaresearch, reporting guidelines and quality scoring tools are not frequently used for self-reporting purposes in radiomics research [27], similar to the findings in another work on an AI reporting guideline for medical imaging [28] and to those related to other general reporting guidelines. This might be related to limited encouragement and endorsement of reporting guidelines by journals for several reasons and to some reluctance of authors to show the limited quality [29, 30].

Developed with a modified Delphi method and published in the first half of 2023, the CheckList for EvaluAtion of Radiomics research (CLEAR) is a 58-item checklist designed specifically for radiomics research [23] (Table 1). It has been endorsed by the European Society of Radiology and the European Society of Medical Imaging Informatics (EuSoMII).

**Table 1:** Items of CLEAR checklist [23]

**Section**	**No.**	**Item**
**Title**		
	1	Relevant title, specifying the radiomic methodology
**Abstract**		
	2	Structured summary with relevant information
**Keywords**		
	3	Relevant keywords for radiomics
**Introduction**		
	4	Scientific or clinical background
	5	Rationale for using a radiomic approach
	6	Study objective(s)
**Method**		
*Study design*	7	Adherence to guidelines or checklists (*e.g.*, CLEAR checklist)
	8	Ethical details (*e.g.*, approval, consent, data protection)
	9	Sample size calculation
	10	Study nature (*e.g.*, retrospective, prospective)
	11	Eligibility criteria
	12	Flowchart for technical pipeline
*Data*	13	Data source (*e.g.*, private, public)
	14	Data overlap
	15	Data split methodology
	16	Imaging protocol (*i.e.*, image acquisition and processing)
	17	Definition of non-radiomic predictor variables
	18	Definition of the reference standard (*i.e.*, outcome variable)
*Segmentation*	19	Segmentation strategy
	20	Details of operators performing segmentation
*Pre-processing*	21	Image pre-processing details
	22	Resampling method and its parameters
	23	Discretization method and its parameters
	24	Image types (*e.g.*, original, filtered, transformed)
*Feature extraction*	25	Feature extraction method
	26	Feature classes
	27	Number of features
	28	Default configuration statement for remaining parameters
*Data preparation*	29	Handling of missing data
	30	Details of class imbalance
	31	Details of segmentation reliability analysis
	32	Feature scaling details (*e.g.*, normalization, standardization)
	33	Dimension reduction details
*Modeling*	34	Algorithm details
	35	Training and tuning details
	36	Handling of confounders
	37	Model selection strategy
*Evaluation*	38	Testing technique (*e.g.*, internal, external)
	39	Performance metrics and rationale for choosing
	40	Uncertainty evaluation and measures (*e.g.*, confidence intervals)
	41	Statistical performance comparison (*e.g.*, DeLong’s test)
	42	Comparison with non-radiomic and combined methods
	43	Interpretability and explainability methods
**Results**		
	44	Baseline demographic and clinical characteristics
	45	Flowchart for eligibility criteria
	46	Feature statistics (*e.g.*, reproducibility, feature selection)
	47	Model performance evaluation
	48	Comparison with non-radiomic and combined approaches
**Discussion**		
	49	Overview of important findings
	50	Previous works with differences from the current study
	51	Practical implications
	52	Strengths and limitations (*e.g.*, bias and generalizability issues)
**Open Science**		
*Data availability*	53	Sharing images along with segmentation data [n/e]
	54	Sharing radiomic feature data
*Code availability*	55	Sharing pre-processing scripts or settings
	56	Sharing source code for modeling
*Model availability*	57	Sharing final model files
	58	Sharing a ready-to-use system [n/e]

*n/e*, Not essential

One of the major strengths that distinguishes CLEAR from the other radiomic guidelines is its systematic and transparent development methodology [31]. The CLEAR checklist can be accessed at https://clearchecklist.github.io/clear_checklist/CLEAR.html[23]. The overall aim of CLEAR is to improve the completeness and transparency of radiomics research presentation. Consulting this document at the beginning of radiomics research planning may also be useful in improving study design. On the other hand, it should be used after the research is completed for better documentation of the study methods during manuscript preparation. Similarly, it is useful not only for authors but also for reviewers in the peer review process. However, CLEAR is intended to be a reporting guideline rather than a quality assessment tool. For the latter task, the METhodological RadiomICs Score (METRICS), also endorsed by EuSoMII, has been developed to provide a structured approach for *post hoc* research evaluation [24].

The value of explanation and elaboration documents for reporting guidelines has been previously highlighted [32]. Well-known checklists have also stressed the importance of publishing explanation and elaboration documents [33–35]. Lack of explanation and elaboration documents may result in divergences of opinion over the meaning of specific items, which might be an obstacle to the implementation of reporting checklists [27, 28, 30].

Here, we present an explanation and elaboration paper for CLEAR, including positive examples for each item from published original articles on radiomics. The resulting collection, CLEAR Explanation and Elaboration with Examples (CLEAR-E3), is intended to improve CLEAR’s adoption and dissemination, which will ultimately contribute to more transparent, complete, and accurate reporting of radiomics research.

## Development of CLEAR-E3

### Contributor recruitment and task definition

The project was proposed by the lead author (B.K.) and members recruited among the EuSoMII Radiomics Auditing Group through an open call.

Each contributor was assigned to 4 to 5 CLEAR items, with specific instructions aimed at ensuring diversity, relevance, and adherence to predefined open-access standards. All contributors were instructed to provide at least two distinct examples for each item, encompassing different aspects and sourced from various papers, without restriction to a specific database (*e.g.*, PubMed, Scopus, and/or Web of Science). The focus was on reporting practices in accordance with CLEAR, rather than assessing the overall methodological quality of the selected articles. Additionally, they were encouraged to explore examples beyond textual content, including tables and figures if necessary. Examples were required to be sourced from articles with an appropriate Creative Commons (CC) license, allowing the reuse of the material with proper citation and attribution of the work. To prevent any misuse of licensed work or copyright infringement, all CC license attributes of the source papers were carefully evaluated for inclusion in this work. Each example was required to be accompanied by a brief explanation and elaboration paragraph that incorporates the theoretical basis of the CLEAR item with references to relevant literature when applicable.

### Presentation of examples

Adhering to the principles of the CC license type associated with the referenced paper, certain examples were presented verbatim, while others were modified for enhanced clarity, with omitted text indicated by bracketed ellipses (*i.e.*, “[…]”). Citations in the original CLEAR item explanations and examples were intentionally excluded to avoid potential confusion. To avoid any potential copyright infringement, one exemption for excluding these citations was given to papers with a “nonderivative” attribute in their CC license. These papers were carefully chosen to include excerpts that do not contain any citations.

### Finalized CLEAR-E3

**Fig. 1:**
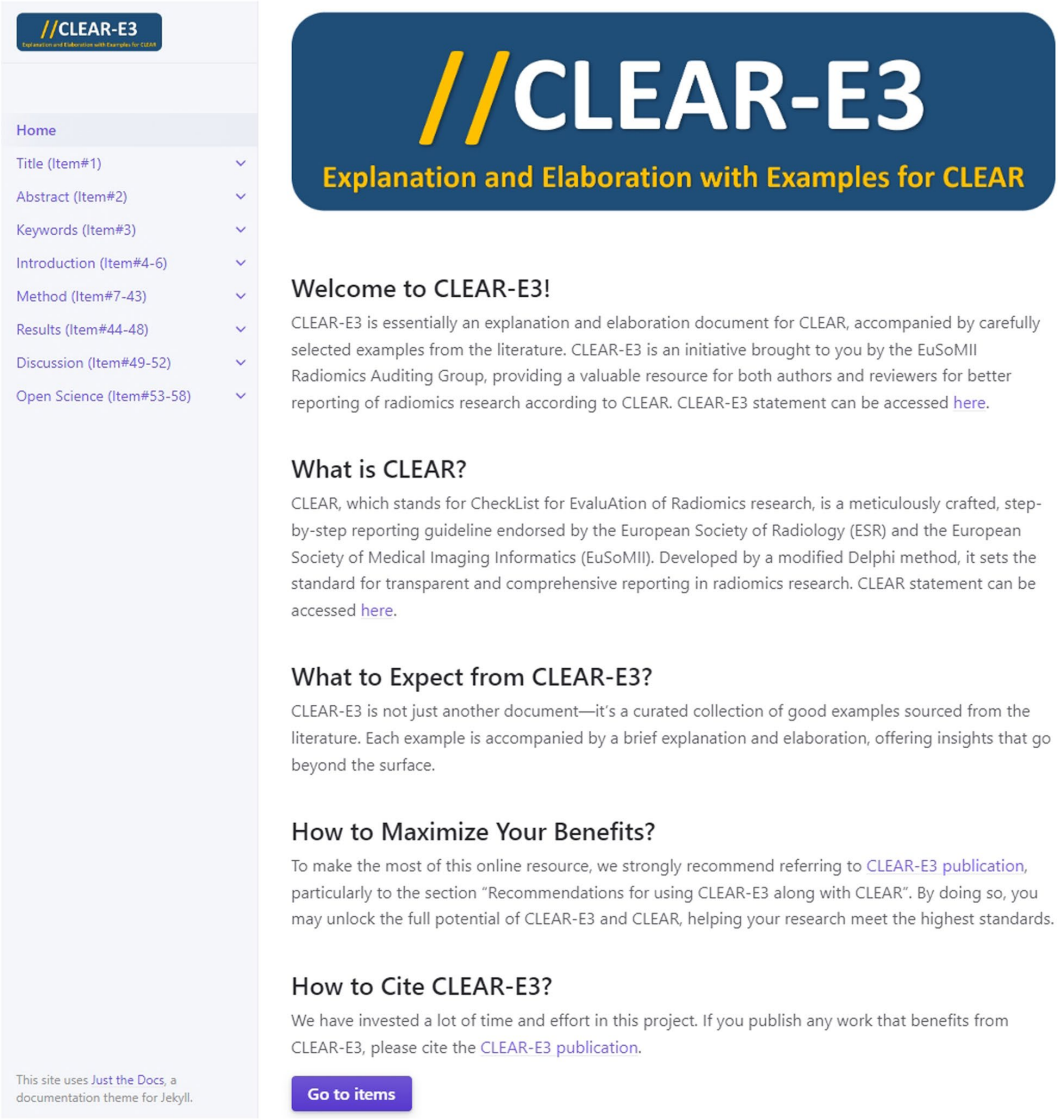
CLEAR-E3’s interactive website. The entire website is accessible at https://radiomic.github.io/CLEAR-E3/

**Fig. 2:**
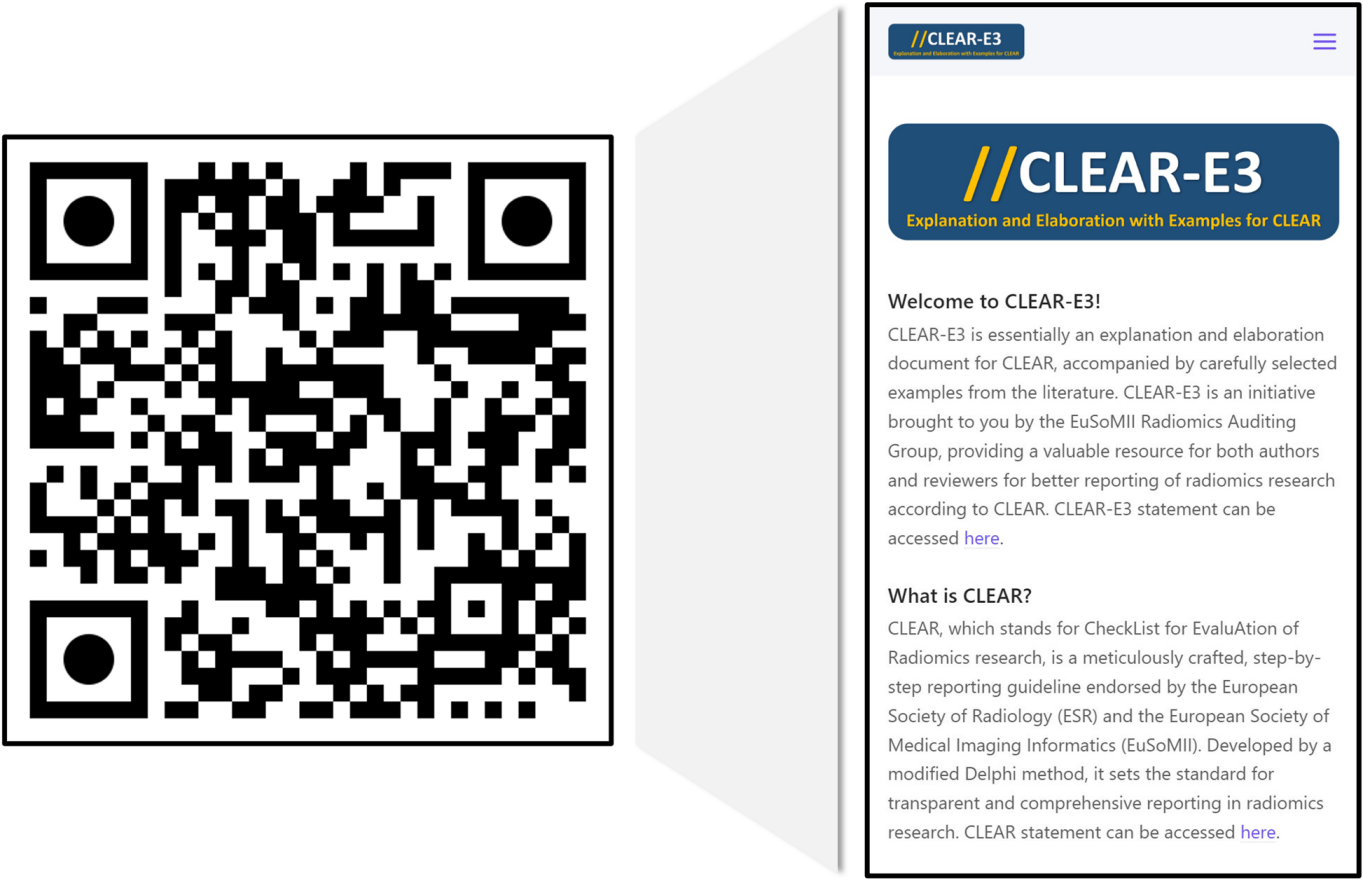
QR code to access the mobile-friendly website

**Fig. 3:**
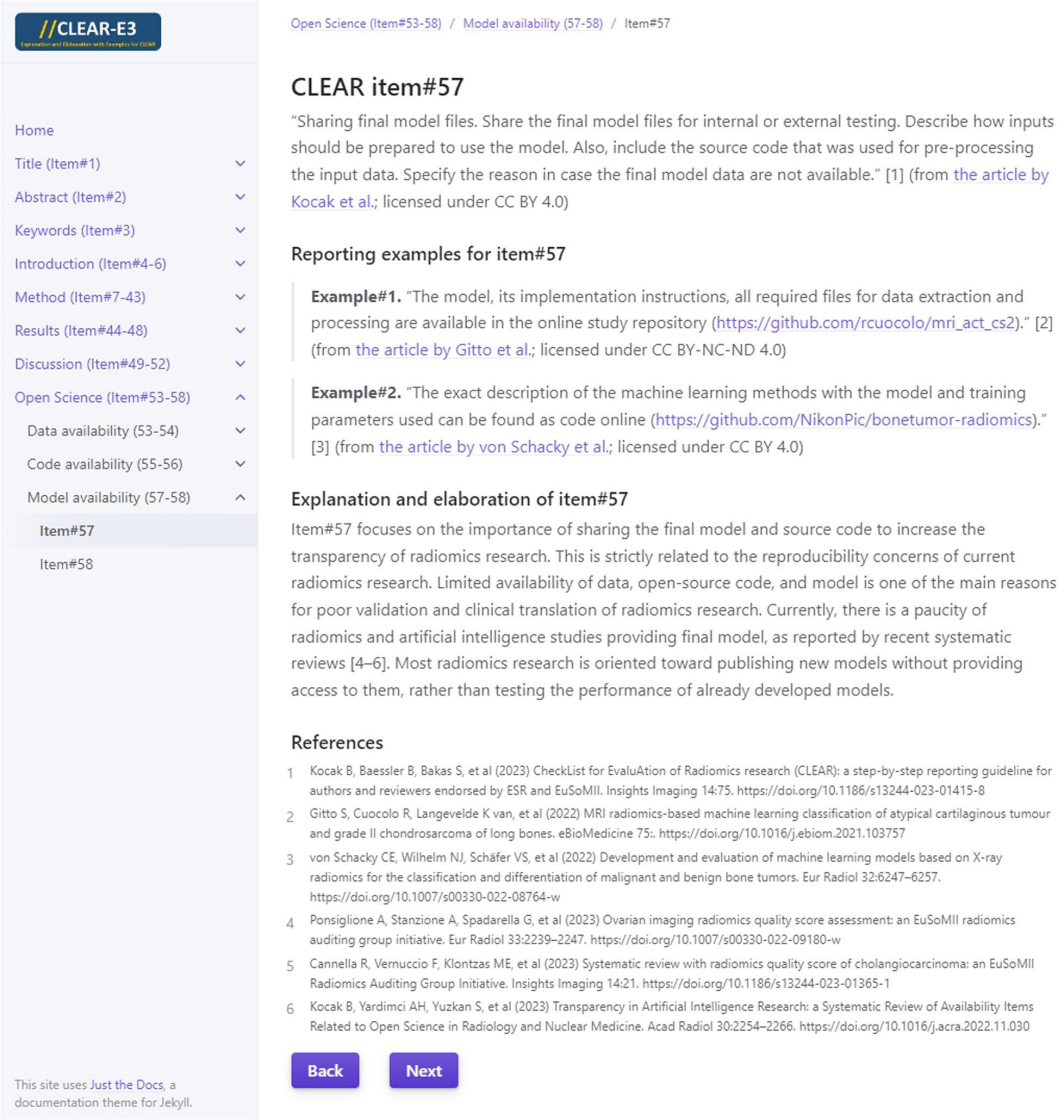
Explanation and elaboration with selected examples from literature for item#57 on the CLEAR-E3’s interactive website. The entire web page is accessible at https://radiomic.github.io/CLEAR-E3/

To allow easier navigation, the resulting CLEAR-E3 can be accessed at https://radiomic.github.io/CLEAR-E3/. Homepage of the CLEAR-E3’s interactive website is shown in Fig. 1. QR code displayed in Fig. 2 can be used to access the mobile-friendly website of CLEAR-E3. Figure 3 presents an example from CLEAR-E3.

## Recommendations for using CLEAR-E3 along with CLEAR

**Fig. 4:**
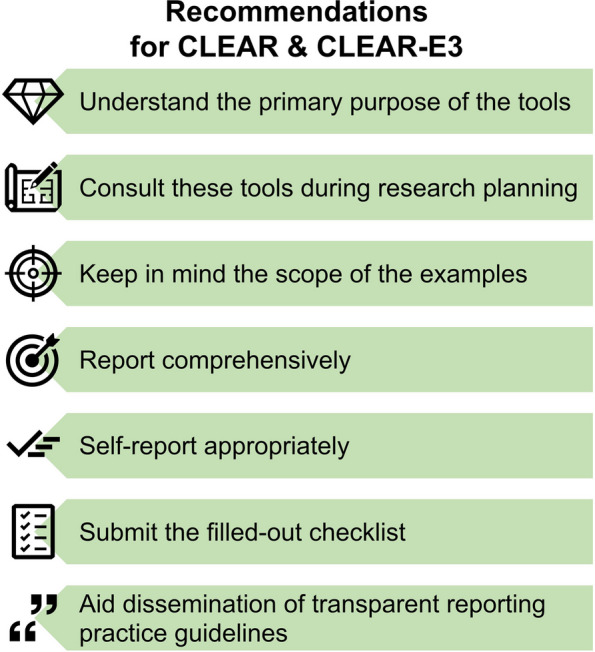
Recommendations for using CLEAR-E3 along with CLEAR

The CLEAR-E3 team strongly advises that users of CLEAR and CLEAR-E3 carefully consider the following recommendations (Fig. 4).

### Understand the primary purpose of the tools

Both CLEAR and CLEAR-E3 are tools designed to improve thoroughness and clarity when reporting radiomics research. Conversely, these documents are not intended to judge the quality of the methods used in the radiomic papers. For the latter case, the use of quality assessment tools (*i.e.*, METRICS) is strongly recommended [24]. Furthermore, CLEAR-E3 is intended as a complement and not a substitute for CLEAR. We recommend using the CLEAR-E3 along with CLEAR whenever deemed necessary.

### Consult these tools during research planning

Consulting this document when initially designing radiomics research could help researchers in systematically gathering important information, as well as prevent issues that limit the downstream applicability and reliability of the radiomics pipeline.

### Keep in mind the scope of the examples

For each CLEAR item, we aimed to present at least two examples from published articles. The examples in CLEAR-E3 are intended to represent appropriate reporting for the corresponding item. Although CLEAR-E3 is expected to offer valuable guidance, by no means do we believe that optimal reporting of radiomics research is solely limited to the examples provided in CLEAR-E3. Furthermore, while these included examples from the literature represent good reporting practice for the corresponding item, this does not guarantee overall CLEAR adherence or methodological quality of the referenced study as a whole.

### Report comprehensively

Authors should adhere to all of the CLEAR items and not just partially. Also, reporting should cover as much of what is mentioned in an item definition as possible instead of just partially. If it is not possible to report all the important information, *e.g.*, due to the word limit, researchers can summarize the important information in a table or figure and/or add more information to the supplementary material. Although not mandatory, reporting in line with the order of CLEAR items would make it easier to identify the key information being reported such as in systematic reviews.

### Self-report appropriately

When self-reporting items using the CLEAR checklist, “not applicable” should always be used appropriately, particularly when the item definition hints at its use by containing an “if applicable” statement. If an item does not include an “if applicable” statement then it should be conclusively answered with “yes” or “no” (*i.e.*, reported or not reported), as this may otherwise lead to incorrect judgments among evaluators [27, 28].

### Submit the filled-out checklist

Authors of radiomics research studies should include a completed CLEAR checklist with their submission to help the editorial process, the peer reviewers, and finally the readers and systematic reviewers of these studies. We suggest using a section heading or its abbreviation along with a paragraph number (*e.g.*, for the second paragraph of the introduction; Intro p2; for the third paragraph of methods, Met p3,) instead of line or page numbers, since changing the page or line number during or after the publishing process can make it hard to keep track of them.

### Aid dissemination of transparent reporting practice guidelines

To facilitate dissemination and appropriate use of CLEAR, we recommend that authors refer this open-access CLEAR-E3 publication along with the original CLEAR statement. Moreover, we recommend reviewers assess whether authors have appropriately cited these publications in their articles to reinforce the significance of and to help disseminate transparent reporting practices. When reporting articles, it is strongly advised to add the following statement: “This study was prepared in accordance with CLEAR and CLEAR-E3,” with citations to CLEAR and CLEAR-E3 publications.

## Final remarks

While CLEAR was intended to enhance the quality of reporting in radiomics research, its actual impact will only become apparent in the future. While formal evaluation studies are warranted, our focus has been on developing strategies to improve the appropriate use of CLEAR within the radiomics community. This initiative has been epitomized in the international collaborative effort to create CLEAR-E3, aimed to facilitate the understanding of CLEAR items. A key difference of CLEAR-E3 from similar previous explanation and elaboration papers is its website. This interactive website greatly improves navigation and eases the use of CLEAR-E3, given the high number of items in CLEAR. Furthermore, we offered collective recommendations for the effective use of CLEAR-E3 as a complement to the CLEAR statement. We welcome feedback from the readers to continuously refine and enhance CLEAR and CLEAR-E3, ensuring their continued efficacy in advancing reporting standards in radiomics research.

## Data Availability

All data and materials are shown in the submission.

## Abbreviations

CC: Creative Commons
CLEAR: CheckList for EvaluAtion of Radiomics research
CLEAR-E3: Explanation and Elaboration with Examples for CLEAR
EuSoMII: European Society of Medical Imaging Informatics
METRICS: METhodological RadiomICs Score
